# The Effects of Mechanical Loading on Tendons - An *In Vivo* and *In Vitro* Model Study

**DOI:** 10.1371/journal.pone.0071740

**Published:** 2013-08-19

**Authors:** Jianying Zhang, James H-C. Wang

**Affiliations:** MechanoBiology Laboratory, Departments of Orthopaedic Surgery, Bioengineering, Mechanical Engineering and Materials Science, and Physical Medicine and Rehabilitation, University of Pittsburgh, Pittsburgh, Pennsylvania, United States of America; University of Notre Dame, United States of America

## Abstract

Mechanical loading constantly acts on tendons, and a better understanding of its effects on the tendons is essential to gain more insights into tendon patho-physiology. This study aims to investigate tendon mechanobiological responses through the use of mouse treadmill running as an *in vivo* model and mechanical stretching of tendon cells as an *in vitro* model. In the *in vivo* study, mice underwent moderate treadmill running (MTR) and intensive treadmill running (ITR) regimens. Treadmill running elevated the expression of mechanical growth factors (MGF) and enhanced the proliferative potential of tendon stem cells (TSCs) in both patellar and Achilles tendons. In both tendons, MTR upregulated tenocyte-related genes: collagen type I (Coll. I ∼10 fold) and tenomodulin (∼3–4 fold), but did not affect non-tenocyte-related genes: LPL (adipocyte), Sox9 (chondrocyte), Runx2 and Osterix (both osteocyte). However, ITR upregulated both tenocyte (Coll. I ∼7–11 fold; tenomodulin ∼4–5 fold) and non-tenocyte-related genes (∼3–8 fold). In the *in vitro* study, TSCs and tenocytes were stretched to 4% and 8% using a custom made mechanical loading system. Low mechanical stretching (4%) of TSCs from both patellar and Achilles tendons increased the expression of only the tenocyte-related genes (Coll. I ∼5–6 fold; tenomodulin ∼6–13 fold), but high mechanical stretching (8%) increased the expression of both tenocyte (Coll. I ∼28–50 fold; tenomodulin ∼14–48 fold) and non-tenocyte-related genes (2–5-fold). However, in tenocytes, non-tenocyte related gene expression was not altered by the application of either low or high mechanical stretching. These findings indicate that appropriate mechanical loading could be beneficial to tendons because of their potential to induce anabolic changes in tendon cells. However, while excessive mechanical loading caused anabolic changes in tendons, it also induced differentiation of TSCs into non-tenocytes, which may lead to the development of degenerative tendinopathy frequently seen in clinical settings.

## Introduction

Mechanical loading in the form of exercise produces multiple health benefits for the musculoskeletal system [1]. For example, tendons, along with muscles and bones, are strengthened by exercise, as indicated by increases in cross-sectional area [2], tendon stiffness [3], and tensile strength [4], [5]. This tendon strengthening occurs because exercise leads to anabolic responses of tendons such as increase in the formation of type I collagen in peritendinous tissue, as shown by microdialysis measurements [6], [7].

One indication of this active tendon response to mechanical loading is the high expression of the anabolic growth hormone gene, IGF-1 [8], [9]. IGF-1 is of particular interest because its Eb isoform, also known as mechano-growth factor (MGF), may be a key component of the mechanism that translates mechanical loads into cellular biological changes. An increase in MGF expression was observed in the Achilles tendons of rats subjected to concentric, eccentric and isometric training by electric stimulation of the sciatic nerve [9]. However, how various mechanical loading conditions, such as treadmill running, affects MGF gene expression in tendons remains undefined.

There is little doubt that cells in tendons are responsible for such mechanobiological responses of tendons. It has long been thought that the majority of cells in tendons are tenocytes, the resident cells responsible for the maintenance and repair of tendons. However, a new type of tendon cells, termed tendon stem/progenitor cells (TSCs), has been recently identified in humans [10], mice [10], [11], rats [12], [13], and rabbits [14]. TSCs differ from tenocytes in that they form colonies in culture and can self-renew; additionally, TSCs have multi-differentiation potential, which enables them to differentiate into tenocytes and non-tenocytes [10], [14], including adipocytes, chondrocytes, and osteocytes under appropriate conditions. For example, in response to low mechanical loading *in vitro*, rabbit TSCs (rTSCs) undergo tenogenic differentiation by expressing tenocyte-related genes, including collagen type I, tenomodulin, and tenascin C; however, under high mechanical loading *in vitro*, rTSCs also express non-tenocyte related genes, including PPARγ, Sox9, and Runx2, the markers for adipocytes, chondrocytes, and osteocytes, respectively [15].

In animal studies, the expression of procollagen III and tenascin C in loaded tendons was shown to be upregulated compared to non-loaded control tendons [16]. In addition, the genes of TGF-β1, collagen I, and collagen III also increased their expression in rat tendons in response to physical training [17]. The increase of metabolic activities in tendons subjected to mechanical loading conditions such as exercise indicates that the tendon does not merely act as a passive connector between muscles and bone, but rather actively responds to mechanical loading placed on it [18], [19]. However, whether different amounts of mechanical loading on tendons *in vivo* induce differential expression of both tenocyte and non-tenocyte related genes, remains to be determined.

Therefore, this study was designed to define the effects of mechanical loading on tendons; specifically, in terms of tendons’ gross response, MGF gene expression, characteristics (proliferation and stem cell marker expression) of TSCs, and expression of tendinous and non-tendinous tissue related genes. To that end, we used the mouse treadmill running model to apply moderate and intensive loads on mouse tendons, then assessed expression of MGF, and tenocyte and non-tenocyte related genes in loaded tendons and control tendons without treadmill running. We also used a well-established *in vitro* model [20] to apply low and high mechanical loads to tendon cells (TSCs and tenocytes) to assess cellular gene expression patterns for comparison with the *in vivo* study.

## Materials and Methods

### Ethics Statement

The University of Pittsburgh IACUC approved all experimental protocols using mice including the treadmill running and collection of tendon samples.

### Mouse Treadmill Running Model

A total of 18 C57BL/6J female mice (2.5 months old) obtained from The Jackson Laboratory (Bar Harbor, ME) were divided into three groups: moderate treadmill running group (MTR), intensive treadmill running group (ITR), and cage control group (Cont), with 6 mice in each group.

In the first week, all 12 mice in both the MTR and ITR groups received training for treadmill running (Exer-6M Open Treadmill; Columbus Instruments, Columbus, OH) at 13 m/min, 15 min/day, and 5 days/week. Following this training period, mice in the MTR group ran at the same speed for 50 min/day, 5 days/week, and 3 weeks in total. On the other hand, mice in the ITR group ran at the same speed for 3 hrs/day, 4 hrs/day, and 5 hrs/day for 5 days in the second, third, and fourth weeks, respectively. The mice in control group were allowed to move freely in cages during treadmill running experiments.

After the end of treadmill running, the mice were sacrificed, and their patellar and Achilles tendons was inspected and subsequently documented by photography. After this, the mice were used for cellular and gene expression analyses.

### Isolation of Tendon Cells from Mechanically Loaded Tissues

Using a previously published method [14], mouse TSCs within the tendon tissues of various groups were isolated for characterization. Briefly, the patellar and Achilles tendons were harvested by detaching them from their muscular and bony attachments. Three mice were used from each group and care was taken to make sure that only tendinous tissues were collected. After removing the paratenons, both patellar and Achilles tendon samples from one mouse leg each were individually used for tendon cell culture and characterization. In total, six independent tendon cell cultures were established from six tendon samples per group (*i.e.* patellar and Achilles tendons from three mice). The remaining three mice were used for gene expression analysis by quantitative RT-PCR (qRT-PCR). Tendon samples from both control and experimental mice were weighed and cut into small pieces. Then, patellar and Achilles tendon samples from the same mouse were immersed separately in 0.5 ml of phosphate-buffered saline with 3 mg/ml collagenase type I (Worthington Biochemical Corporation, Lakewood, NJ) and 4 mg/ml dispase (StemCell Technologies Inc., Vancouver, BC, Canada) at 37°C for 1 hr. The cell suspensions were centrifuged at 3,000 rpm for 15 min to obtain cell pellets, which were re-suspended in growth medium consisting of Dulbecco’s modified Eagle’s medium (DMEM; Lonza, Walkersville, MD) supplemented with 20% fetal bovine serum (FBS, Atlanta Biologicals Lawrenceville, GA) and 1% penicillin and streptomycin (Atlanta Biologicals, Lawrenceville, GA). The collagenase-dispase solution was discarded. Cell suspension from the patellar and Achilles tendons of the same mouse was plated separately in a 6-well culture plate. The cells were observed for colony development after 10 to 14 days in primary culture and the morphology of cells was then observed under a microscope. An automated cell counter (Cellometer, Nexcelom, Lawrence, MA) was used to determine total cell numbers after the cells were detached using 0.05% trypsin at about 90% confluence.

Trypsin was used to detach each cell colony visualized under a microscope. A micropipette was used to collect each individual detached cell colony and then transferred to individual wells of a 24-well plate for further culture. After removal of cell colonies, tenocytes were left in culture plates for additional experiments. These cells were elongated in shape and cultured in DMEM containing 10% FBS, and 1% penicillin and streptomycin.

### TSC Proliferation and Stem Cell Marker Expression

TSCs at passage 1 were seeded in a 6-well plate at the density of 3 × 10^4^/well and cultured in growth medium (DMEM+20% FBS) for 5 days. To measure cell proliferation, we determined the population-doubling time (PDT) of TSCs, which is expressed as log_2_[Nc/N_0_], where N_0_ is the total number of cells seeded initially, and Nc is the total number of cells at confluence [14].

To determine stem cell marker expression in TSCs, we applied immunocytochemistry to detect the expression of nucleostemin (NS), octamer-binding transcription factor 4 (Oct-4), and stage-specific embryonic antigen-1 (SSEA-1). The cells at passage 1 were seeded in a 12-well plate at the density of 1.5 × 10^4^/well, and then cultured in growth medium for 3 days. Paraformaldehyde (4% in PBS) was then applied for 30 min at room temperature to fix the cells. TSCs were treated with 0.1% Triton X-100 in PBS for 30 min and washed three times with PBS for Oct-4 and nucleostemin staining. They were then incubated with goat anti-mouse nucleostemin (1∶350; Neuromics, Edina, MN; Cat. #GT15050), or rabbit anti-mouse Oct-4 (1∶350; Santa Cruze Biotechnology, Santa Cruz, CA, Cat. #9081) overnight at 4°C. Cultures were washed three times with PBS, followed by application of Cy3-conjugated donkey anti-rabbit IgG antibodies (1∶500 for Oct-4, Millipore, Billerica, MA; Cat. #AC182C), or Cy3-conjugated Donkey anti-goat IgG antibodies (1∶500 for nucleostemin, Millipore, Billerica, MA; Cat. #AC180C) for 2 hrs at room temperature. Staining for SSEA-1 was performed by blocking fixed cells with 2% mouse serum for 1 hr and incubating with FITC-conjugated mouse anti-SSEA-1 (1∶250, Chemicon International, Temecula, CA; Cat. # MAB4301X) for 3 hrs at room temperature. After washing cells with PBS, stained cells were observed under fluorescence microscopy and digital images were recorded.

### Gene Expression Analysis

To characterize mechanobiological response of the tendon to treadmill running, we determined the expression of MGF gene, and the expressions of tenocyte and non-tenocyte related genes (see below) in the tendon tissues using qRT-PCR. For this, both patellar and Achilles tendon samples were collected from 3 mice each from the control and treadmill running groups, weighed, minced, homogenized in lysis buffer (Qiagen, Valencia, CA), and centrifuged at 5,000 g for 15 min to collect the supernatant containing the TSCs. Total RNA was extracted from the tendons using an RNeasy Mini Kit with an on-column DNase I digest (Qiagen, Valencia, CA). First-strand cDNA was synthesized by reverse transcription with SuperScript II (Invitrogen, Grand Island, NY) in a 20 µl reaction containing 1 µg total RNA. The conditions for cDNA synthesis were: 65 °C for 5 min and cooling for 1 min at 4 °C, then 42 °C for 50 min, and finally 72 °C for 15 min. A QIAGEN QuantiTect SYBR Green PCR Kit (Qiagen, Valencia, CA) was used to perform qRT-PCR with 2 µl cDNA (total 100 ng RNA) in a 25 µl PCR reaction mixture using a Chromo 4 Detector (MJ Research, St Bruno, Canada). Previously published mouse-specific primers were used for the following genes: MGF [21], collagen type I [22] and tenomodulin [23] (tenocyte-related genes), lipoprotein lipase (LPL) [24] and PPARγ [25] (two adipocyte-related genes), Sox9 (chondrocyte-related gene) [26], and Runx2 [27] and Osterix [28] (osteocyte-related genes). Glyceraldehyde-3-phosphate dehydrogenase (GAPDH) was used as an internal control [22]. All primers were obtained from Invitrogen (Grand Island, NY) and their sequences are shown in Table 1. PCR reaction conditions were essentially as described previously [21]–[28].

**Table 1:** A list of primers used for gene expression analysis.

Gene	Primer Sequence	Accession Numbers	Reference
MGF	Forward 5′-AGCTGCAAAGGAGAAGGAAAGGAAG-3′	AY878193.1	[21]
	Reverse 5′-GGTGATGTGGCATTTCCTGCT-3′		
Collagen Iα2	Forward 5′-CAACCTGGACGCCATCAAG-3′	NM_007742.3	[22]
	Reverse 5′-CAGACGGCTGAGTAGGGAACA-3′		
Tenomodulin	Forward 5′-TGTACTGGATCAATCCCACTCT-3′	NM_022322.2	[23]
	Reverse 5′-GCTCATTCTGGTCAATCCCCT-3′		
LPL	Forward 5′-AAGCTGGTGGGAAATGATGTGG-3′	NM_008509.2	[24]
	Reverse 5′-CCGTTCTGCATACTCAAAGTTAGG-3′		
PPARγ	Forward 5′-CCACCAACTTCGGAATCAGCT-3′	U01841.1	[25]
	Reverse 5′- TTTGTGGATCCGGCAGTTAAGA-3′		
Sox9	Forward 5′-GAAGTCGCTGAAGAACGGACAAG-3′	NM_011448.4	[26]
	Reverse 5′-GCTGTAGTGAGGAAGGTTGAAGGG-3′		
Runx2	Forward 5′-CCGCACGACAACCGCACCAT-3′	NM_001145920.2	[27]
	Reverse 5′-AGCCACCAAGGCTGGAGTCTT-3′		
Osterix	Forward 5′-AGCGACCACTTGAGCAAACAT-3′	DQ229136.1	[28]
	Reverse-5′GCGGCTGATTGGCTTCTTCT-3′		
GAPDH	Forward 5′-ATGGCCTTCCGTGTTTCCTAC-3′	NM_008084.2	[22]
	Backward 5′-TGATGTCATCATACTTGGCAGG-3′		

The formula 2^−ΔΔCT^, where ΔΔCT = (CT_target_ – CT_GAPDH_)_treated_ – (CT_target_ – CT_GAPDH_)_control_, was used to calculate the relative gene expression levels in tendon cells. CT represents the cycle threshold of each RNA sample. At least three parallel tests were performed to determine standard deviation (SD) of the ΔCT.

### Cell Stretching Experiments *in vitro*

To determine which cell population (TSCs or tenocytes) is responsible for non-tenocyte related gene expression, an established *in vitro* mechanical loading system was used [29]. This system can control tendon cells in such a manner that cell shape and organization mimic those *in vivo*. Therefore, it allows us to closely examine the mechanobiological responses of tendon cells, including cell proliferation and differentiation, under well-controlled, *in vivo*-like mechanical loading conditions.

For all cell stretching experiments, we used TSCs at passage 1 or tenocytes at passage 7, which were isolated from control mice without treadmill running as described under ‘Isolation of tendon cells from mechanically loaded tissues’. These cells were seeded in silicone dishes at a density of 3 ×10^5^/dish and cultured in growth medium with 20% FBS (for TSCs) or 10% FBS (for tenocytes) overnight. Cyclic stretching at 4% and 8% was applied to silicone dishes for 12 hrs. Then RNA was extracted from the three groups of TSCs including un-stretched control, 4% stretched and 8% stretched and cellular gene expression analysis was performed using qRT-PCR essentially as described under ‘gene expression analysis’. Note that the two stretching magnitudes 4% and 8% are so called “clamp-to-clamp” (CTC) engineering strains, not the strains acting on cells, which are smaller than these two CTC strains. The two strain magnitudes are considered to be low and high mechanical loading on tendon cells [15], [29].

### Statistical Analysis

All data are presented as mean ± standard deviation (SD), unless otherwise indicated. For statistical analysis of the data, one-way ANOVA was used, followed by Fisher’s PLSD test for multiple comparisons. A *t*-test was also used for statistical analysis wherever appropriate. P<0.05 (type I error) was considered to indicate significant difference between two groups compared.

## Results

**Figure 1:**
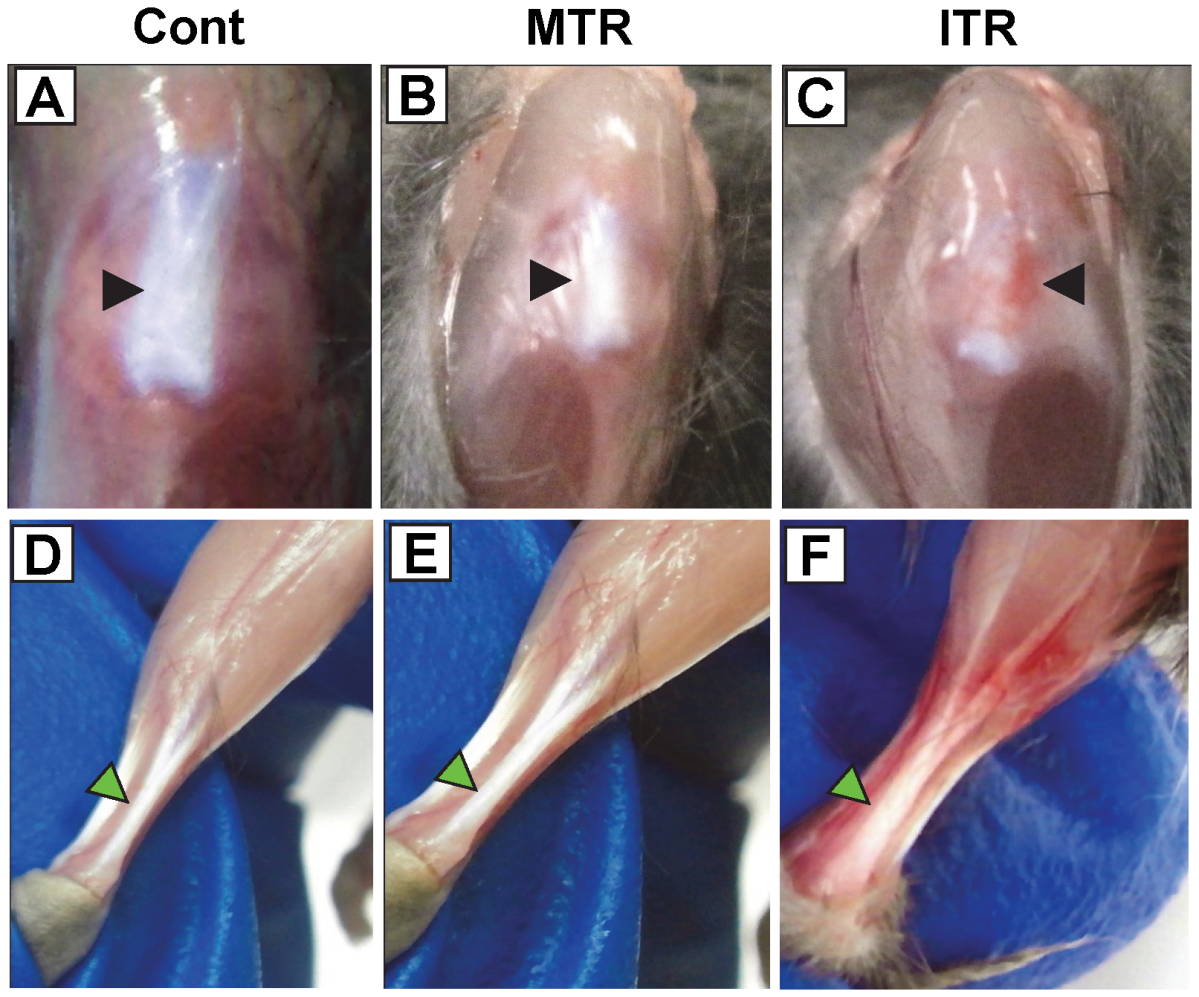
The gross appearance of mouse tendons after treadmill running. **A–C.** Patellar tendons. **D–F.** Achilles tendons. It is evident that without treadmill running (or cage control), both mouse patellar tendon (**A**) and Achilles tendon (**D**) were shiny and avascular (arrows). After moderate treadmill running (MTR), the normal appearance of both patellar and Achilles tendons (**B, E**) apparently remained unchanged (arrows). After intensive treadmill running (ITR), the areas in and around the patellar and Achilles tendons appeared more vascularized, instead of a normal glistening and white appearance, suggesting neo-vascularization (**C, F**; arrows) had occurred.

Normal patellar tendons of control mice without treadmill running were white, shiny (Figure 1A), and could be easily removed from their bony and muscular attachments. Mouse patellar tendons in the MTR group looked similar to those in the control mice (Figure 1B). However, the paratenons of mouse patellar tendons in the ITR group were noticeably more vascular with a tendency to bleed (Figure 1C). The tendon itself appeared softer, stickier, and more elastic. Similar phenomena were also noticed in the mouse Achilles tendons (Figure 1D, E, F).

**Figure 2:**
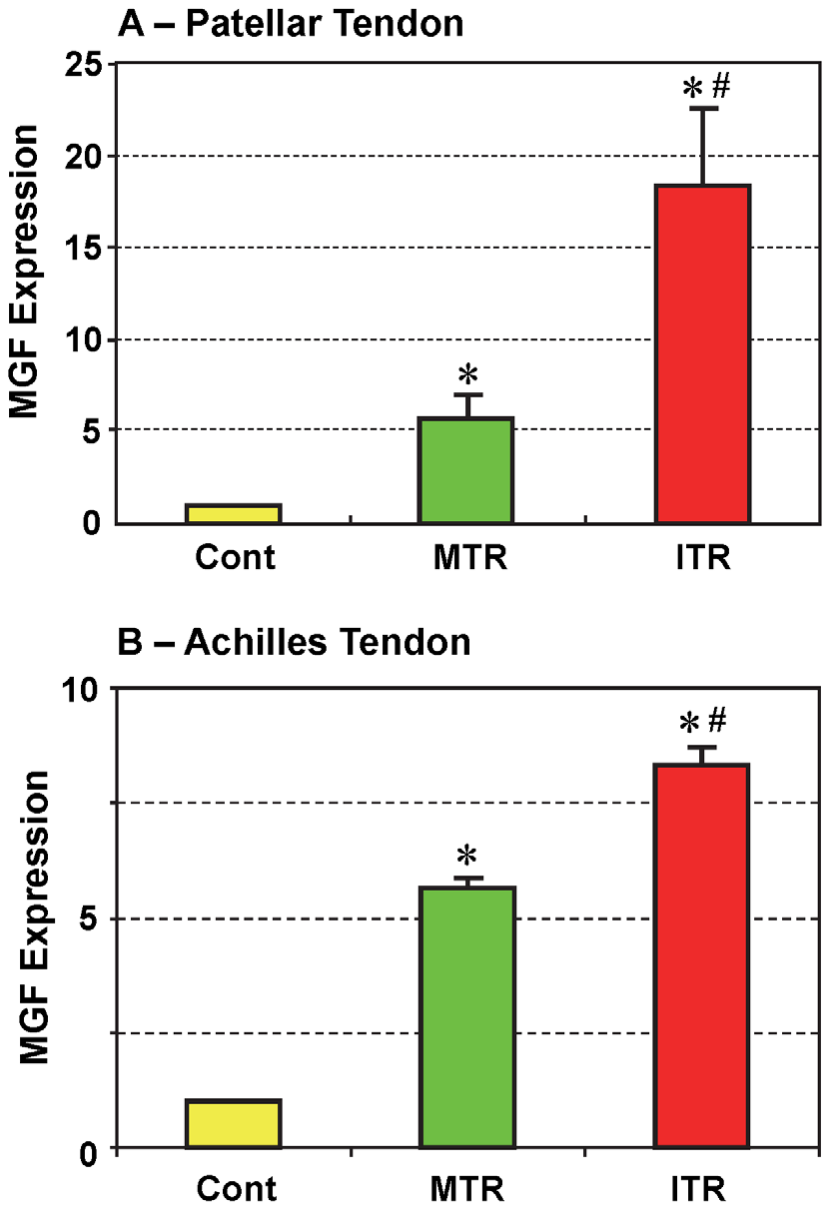
MGF expression in mouse patellar (A) and Achilles (B) tendons in response to moderate treadmill running (MTR) and intensive treadmill running (ITR). Compared to non-running controls, MTR upregulated MGF gene expression in patellar tendons by more than 5-fold, whereas ITR increased the same gene expression by over 17-fold. Similarly, MGF increased 5 times in the Achilles tendons of the MTR group and 7 times in the ITR group (Data are shown as mean ± SD, n  = 6. (*p<0.05 with respect to control; ^#^p<0.05 with respect to MTR).

Higher expression of MGF was found in patellar tendons from all 12 mice that underwent treadmill running (Figure 2A). MGF expression in the ITR group was significantly higher (3 times; p<0.025) than the MTR group, which was approximately 5 times higher (p<0.023) than the control group. Similar results were also found in Achilles tendons with a significant increase in ITR over MTR (Figure 2B; p<6.8E-05). In both tendons, MGF expression in the MTR group was comparable with a 5-fold increase over control while MGF expression in the patellar tendons in ITR group was much higher than that in Achilles tendons. In both patellar and Achilles tendons, MGF expression exhibited a loading (or treadmill running) intensity-dependent increase.

**Figure 3:**
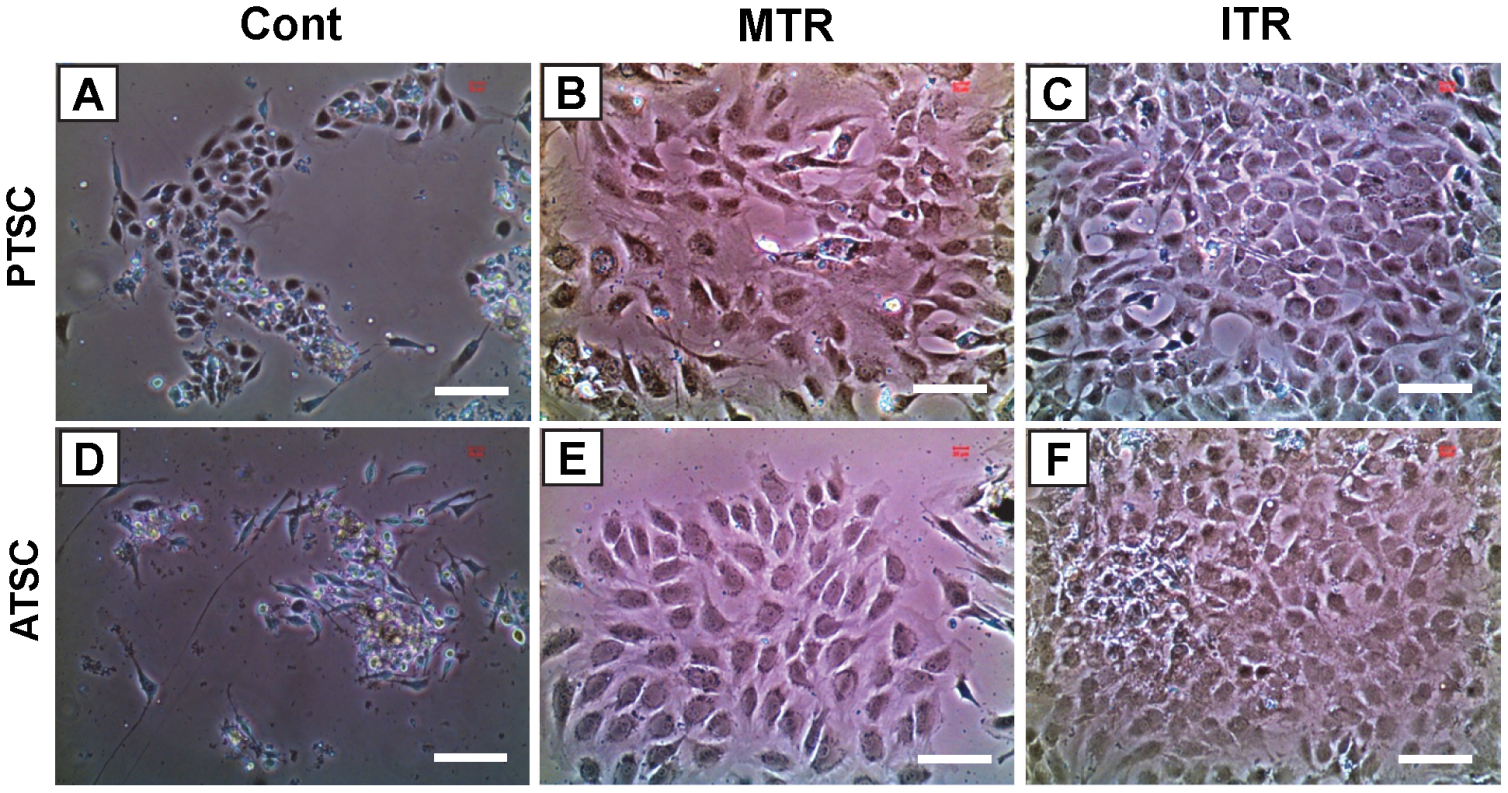
The morphology and colony formation of TSCs isolated from mouse tendons with and without treadmill running. **A–C.** Patellar tendon stem cells (PTSCs); and **D–F.** Achilles tendon stem cells (ATSCs). The stem cells were isolated from the same amounts of patellar and Achilles tendon samples of mice subjected to treadmill running and cage controls. With MTR, and more so with ITR, both PTSCs and ATSCs grew more quickly and formed larger colonies than cage controls. Bars: 100 µm.

**Figure 4:**
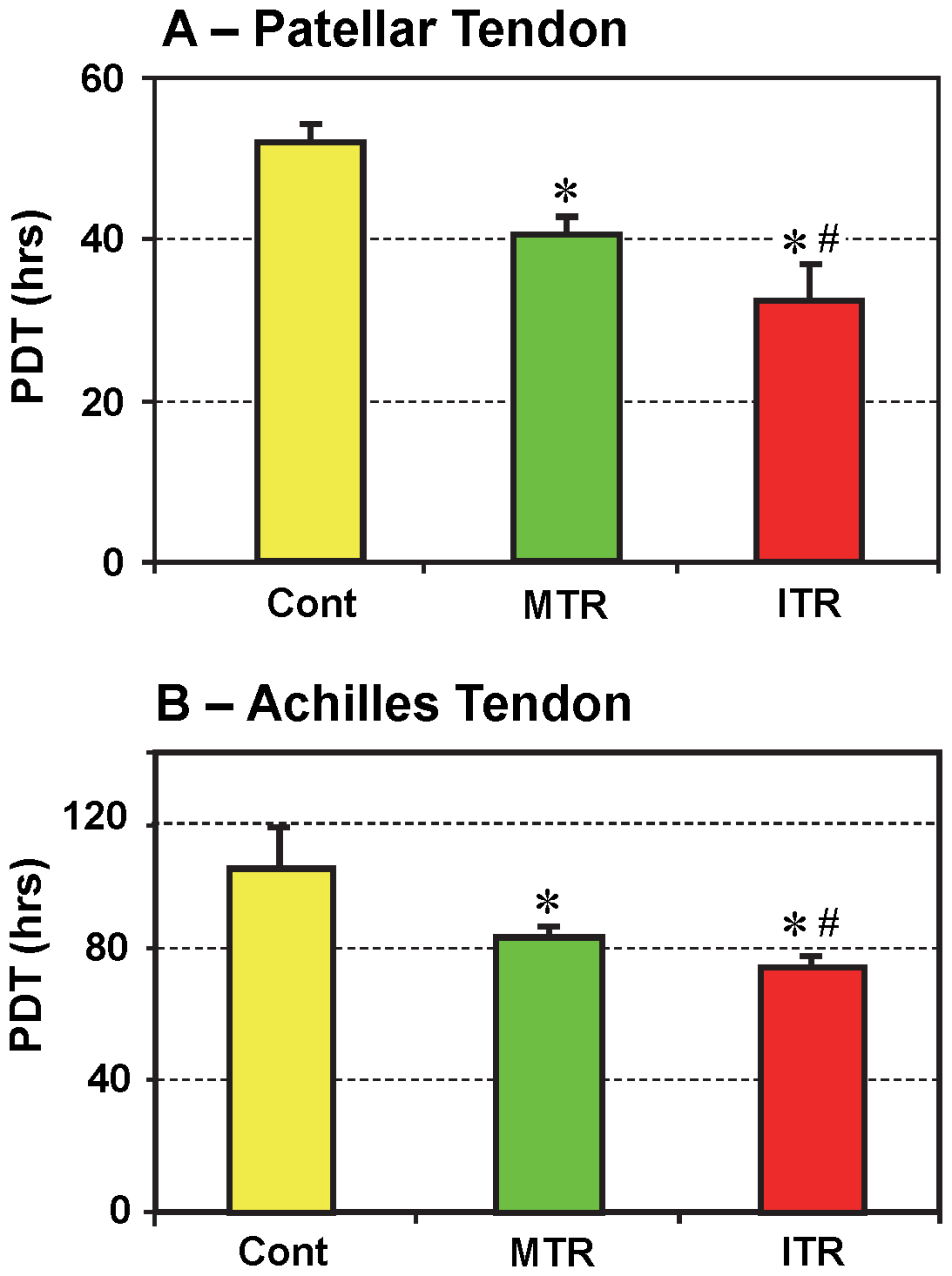
The population doubling time (PDT) of TSCs isolated from mouse tendons. TSCs were seeded in a 6-well plate and cultured to determine PDT. Mouse treadmill running enhanced proliferation of TSCs from patellar tendons (**A**) and Achilles tendons (**B**), as indicated by decreased PDT compared to cage controls. In fact, ITR stimulated cell growth more quickly than MTR (**A, B**) (*p<0.05 with respect to cage control; ^#^p<0.05 with respect to MTR).

The TSCs isolated from mouse patellar tendons (Figure 3A, B, C) and Achilles tendons (Figure 3D, E, F) of all three groups (Cont, MTR, and ITR) showed stem cell morphology, which is a cobblestone shape [14]. However, in both patellar and Achilles tendons, TSCs isolated from the ITR group (Figure 3C, F) formed much larger colonies than those from the MTR group (Figure 3B, E), which were larger than those generated by cells in the control group (Figure 3A, D). These results were also corroborated by quantification of TSCs in both small and large colonies (Table 2, and Table 3). In addition, treadmill running significantly increased TSC proliferation as evidenced by a decrease in population doubling time (PDT). More specifically, cells derived from the MTR group proliferated at a higher rate (p<1.0E-04 for PTSCs; and p<0.013 for ATSCs) than the control group without treadmill running. In addition, TSCs from the ITR group grew significantly quicker (p<0.014 for PTSCs; and p<0.039 for ATSCs) than those from the MTR group (Figure 4).

**Table 2:** Number of TSCs cultured from patellar tendons of mice subjected to treadmill running.

	Numbers of cells in small colonies	Numbers of cells in large colonies
Control	7	113
MTR	34	126
ITR	48	341

**Table 3:** Number of TSCs cultured from Achilles tendons of mice subjected to treadmill running.

	Numbers of cells in small colonies	Numbers of cells in large colonies
Control	4	103
MTR	26	94
ITR	30	349

**Figure 5:**
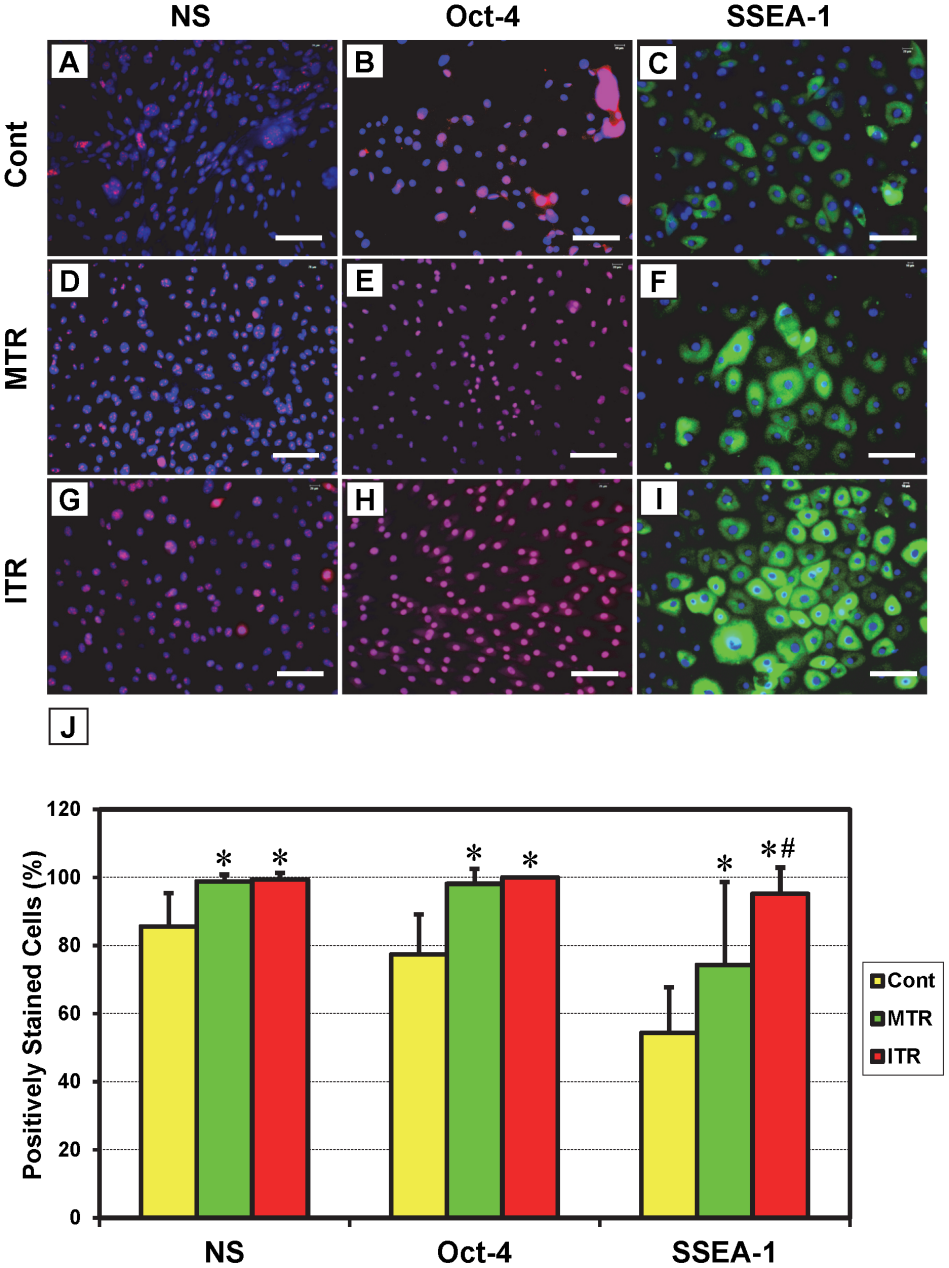
The expression of stem cell markers by patellar tendon stem cells (PTSCs) in response to mouse treadmill running. TSCs were seeded in a 6-well plate, cultured and incubated with goat anti-mouse nucleostemin/Cy3-conjugated donkey anti-goat IgG antibodies (**A, D, G**), rabbit anti-mouse Oct-4/Cy3-conjugated donkey anti-rabbit IgG antibodies (**B, E, H**) or FITC-conjugated mouse anti-SSEA-1 (**C, F, I**). Compared to PTSCs from cage control mice (**A–C**), more cells in MTR (**D–F**) and ITR (**G–I**) groups had increased expression of stem cell markers nucleostemin (NS), Oct-4, and SSEA-1. Note that the extent of the immunostaining on these stem cell markers is apparently running-intensity-dependent. Positively stained cells were also counted to calculate percentage staining (**J**). (*p<0.05 with respect to control; ^#^p<0.05 with respect to MTR). Bar: 100 µm.

**Figure 6:**
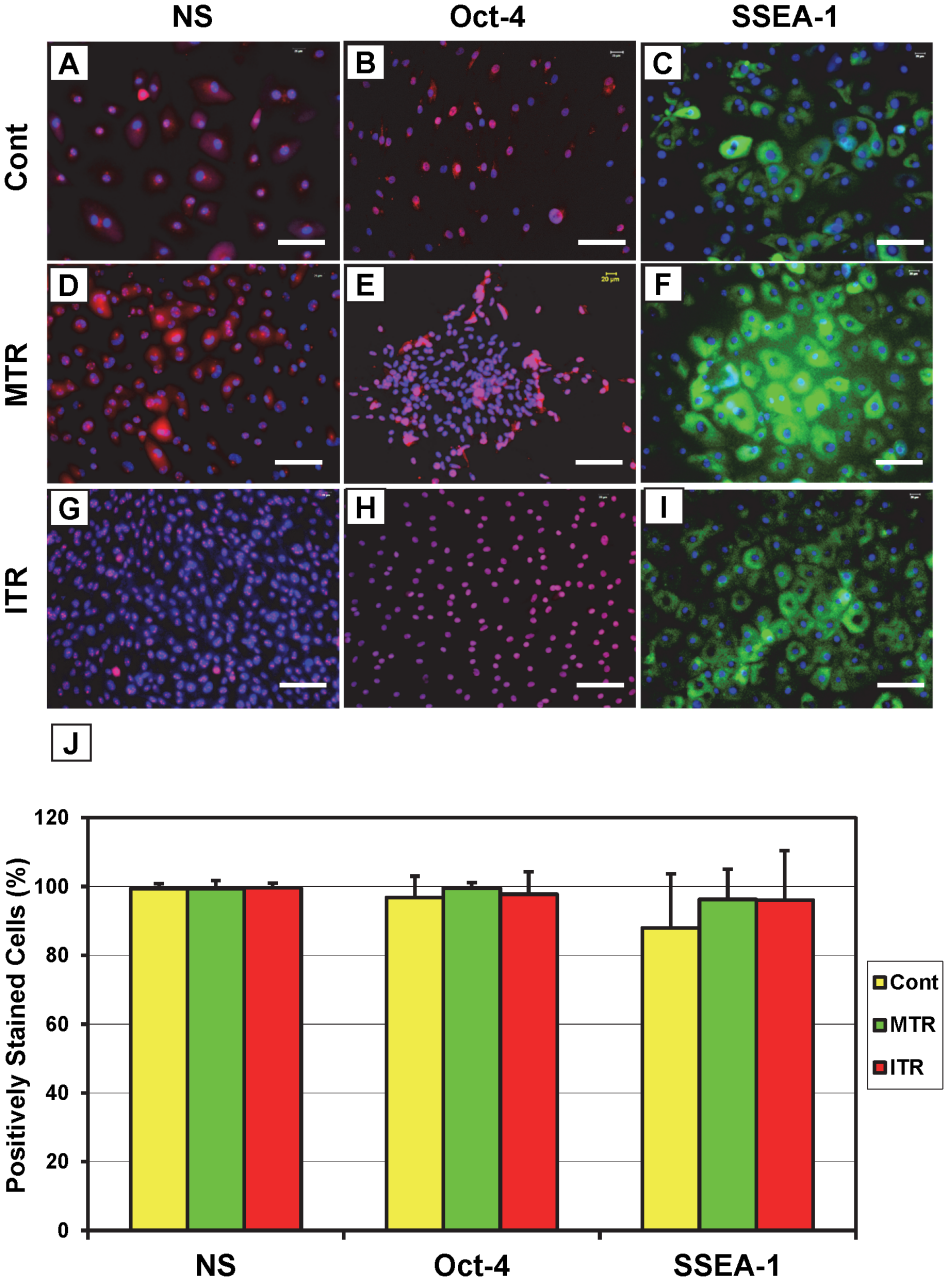
The expression of stem cell markers by Achilles tendon stem cells (ATSCs) in response to mouse treadmill running. TSCs were seeded in a 6-well plate, cultured and incubated with goat anti-mouse nucleostemin/Cy3-conjugated donkey anti-goat IgG antibodies (**A, D, G**), rabbit anti-mouse Oct-4/Cy3-conjugated donkey anti-rabbit IgG antibodies (**B, E, H**) or FITC-conjugated mouse anti-SSEA-1 (**C, F, I**). Compared to ATSCs from cage control mice (**A–C**), more cells in MTR (**D–F**) and ITR (**G–I**) groups increased the expression of stem cell markers nucleostemin (NS), Oct-4, and SSEA-1. It is apparent that the extent of the immunostaining on these stem cell markers is running-intensity-dependent. Positively stained cells were also counted to calculate percentage staining (**J**), which shows no significant difference between the groups. Bar: 100 µm.

The results of cell colony growth and population doubling time (PDT) indicate that mouse treadmill running accelerates TSC proliferation in an intensity-dependent manner. These results were further demonstrated by immunostaining: cells isolated from patellar tendons of the ITR group were positively stained for nucleostemin (Figure 5G), Oct-4 (Figure 5H), and SSEA-1 (Figure 5I) at a higher percentage than those isolated from the MTR group (Figure 5D–F), which was higher than the cage control group (Figure 5A–C). Consistent with these results, significant increases were observed in the numbers of cells that stained positive for NS, Oct-4, and SSEA-1 in the MTR (p<0.002 for NS; p<0.004 for Oct-4; and p<0.023 for SSEA-1) and ITR (p<0.001 for NS; p<0.003 for Oct-4; and p<1.0E-04 for SSEA-1) groups when compared to the cage control (Figure 5J). Between the two running groups, only SSEA-1 staining was significantly higher (p<0.014) in ITR when compared to MTR. However, such increases were minimal in TSCs isolated from Achilles tendons subjected to ITR (Figure 6G–I), MTR (Figure 6D–F), or no treadmill running (Figure 6A–C) and not statistically significant (Figure 6J).

**Figure 7:**
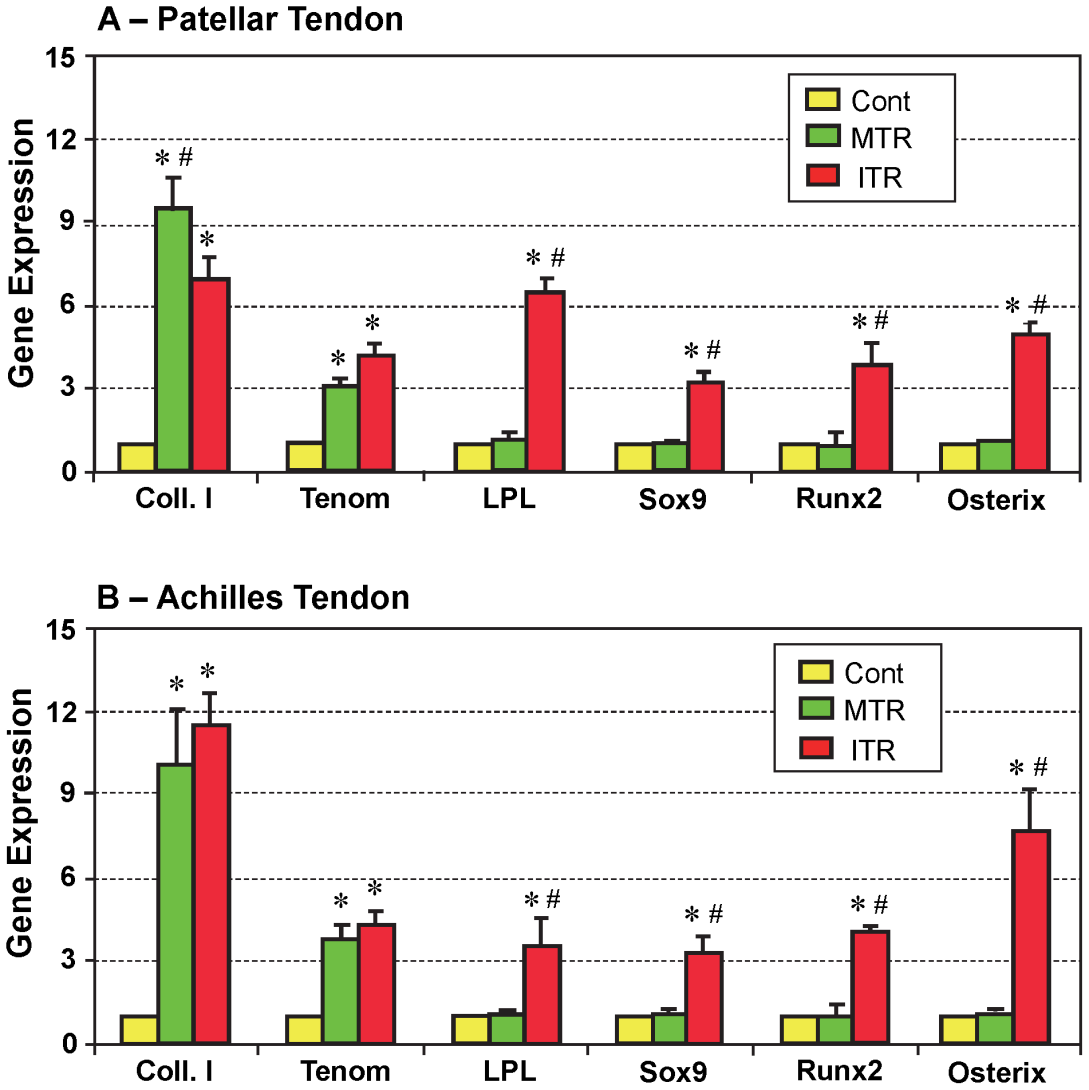
The expression of tenocyte and non-tenocyte related genes in patellar (A) and Achilles (B) mouse tendons in response to treadmill running. Total RNA collected from the tendons of controls and mice in the MTR and ITR groups were subjected to qRT-PCR. As shown, MTR only increased the expression of tenocyte related genes in the two types of tendons (Coll. I, or collagen type I; Tenom or tenomodulin), whereas ITR increased the expression of both tenocyte and non-tenocyte related genes (LPL: a marker for adipocyte; Sox9: a marker for chondrocyte; Runx2 and Osterix: two markers for osteoblasts). (*p<0.05 with respect to the corresponding controls; ^#^p<0.05 with respect to MTR. In A, however, # in Coll. I represents p<0.05 with respect to ITR).

The gene expression in tendon cells isolated from patellar and Achilles tendons of mice that were subjected to MTR and ITR were also analyzed using qRT-PCR. We found that two tenocyte-related genes, collagen type I (Coll. I) and tenomodulin (Tenom), were up-regulated in both patellar (p<0.009 for both Coll. I and Tenom) and Achilles (p<0.002 for Coll. I; and p<0.017 for Tenom) tendons of mice after MTR (Figure 7A, B). However, there was no significant difference in the expression of non-tenocyte related genes, LPL (adipocyte), Sox9 (chondrocyte), and Runx2 and osterix (osteocyte), between the MTR and cage control group. On the other hand, in the ITR group both tenocyte-related and non-tenocyte-related genes were up-regulated in the patellar tendons (p<7.0E-04 for Coll. I; p<0.001 for Tenom; p<2.0E-04 for LPL; p<7.0E-04 for Sox9; p<3.0E-04 for Runx2; and p<0.002 for Osterix) and Achilles tendons (p<5.0E-04 for Coll. I; p<0.002 for Tenom; p<0.004 for LPL; p<0.001 for Sox9; p<0.007 for Runx2; and p<2.0E-04 for Osterix) compared to the tissues from the cage control group (Figure 7A, B). Moreover, the non-tenocyte genes in the ITR group were also significantly up-regulated when compared to the respective MTR groups (patellar tendon: p<3.0E-04 for LPL; p<1.0E-05 for Sox9; p<0.001 for Runx2; and p<0.004 for Osterix. Achilles tendon: p<0.003 for LPL; p<2.0E-04 for Sox9; p<0.034 for Runx2; and p<0.014 for Osterix ). In contrast, Coll. I gene expression in the MTR group was significantly higher (p<0.031) than the ITR group in the patellar tendon.

**Figure 8:**
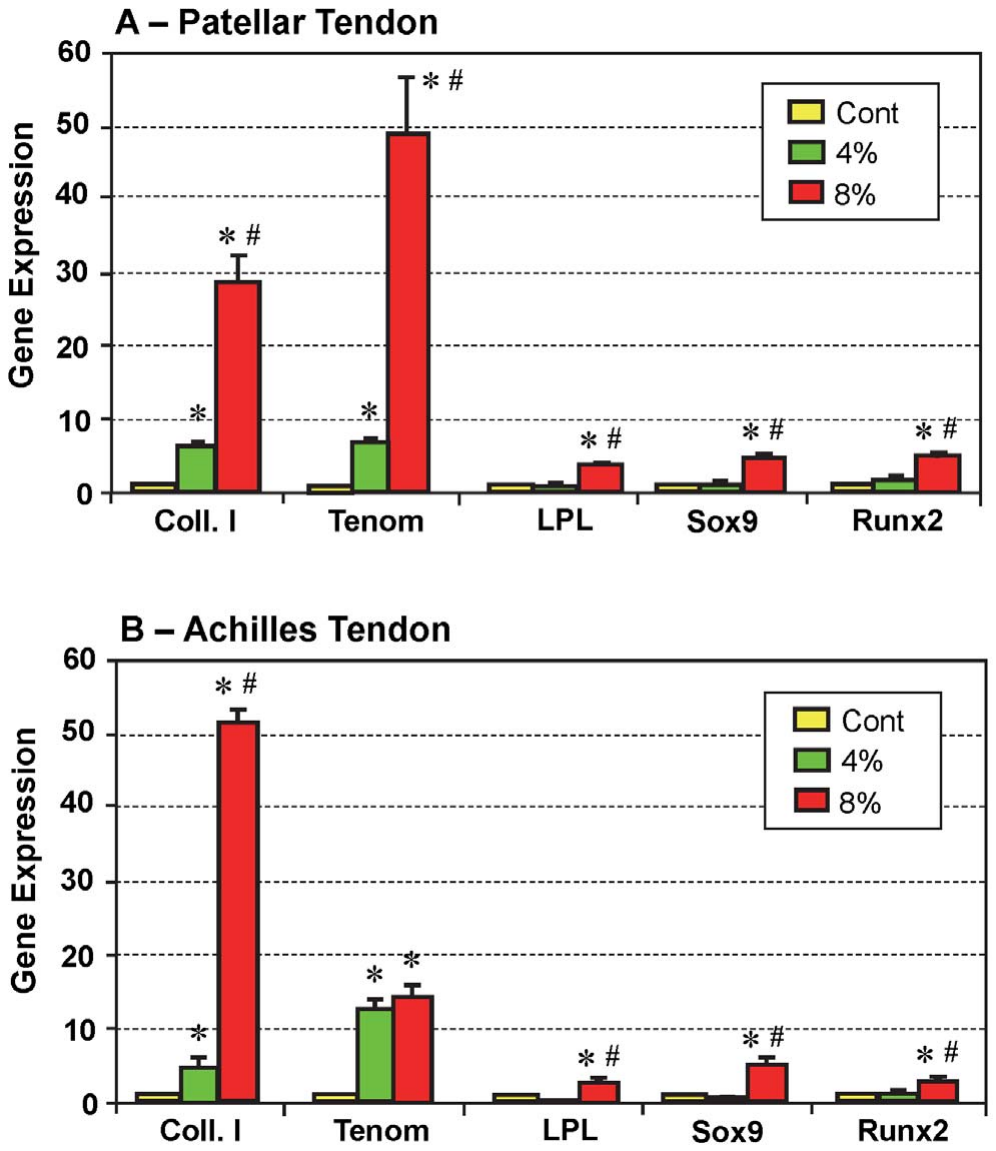
The expression of tenocyte and non-tenocyte related genes in patellar (A) and Achilles (B) TSCs in response to mechanical loading *in vitro*. Total RNA were collected from TSCs stretched to 4% or 8% for qRT-PCR analysis. In PTSCs under low mechanical loading (green, 4% stretching), only those genes related to tenocytes (Coll. I, or collagen type I; Tenom or tenomodulin) were highly expressed, but under high mechanical loading (red, 8% stretching), both tenocyte and non-tenocyte related genes increased their expression. Similar results were obtained for ATSCs in response to low (4%) and high (8%) mechanical loading. Note the different scale in gene expression by PTSCs and ATSCs between the two loading conditions (*p<0.05, with respect to non-loaded cells; ^#^p<0.05 with respect to 4% stretching).

*In vitro* results of mechanical loading experiments also supported the above findings *in vivo*. Specifically, after 4% stretching the expression of tenocyte-related genes was up-regulated in TSCs from both patellar tendons (p<0.011 for Coll. I; and p<0.004 for Tenom) and Achilles tendons (p<0.006 for Coll. I; and p<0.007 for Tenom) (Figure 8A, B). However, there was no significant difference in non-tenocyte-related gene expression in TSCs from the two types of tendons between the 4% stretching and non-loaded control group. On the other hand, in the 8% stretching group, both tenocyte-related and non-tenocyte-related genes in the TSCs from both tendons were significantly up-regulated when compared to controls (Figure 8A, B) (patellar tendon: p<0.02 for Coll. I; p<3.0E-04 for Tenom; p<7.0E-04 for LPL; p<0.005 for Sox9; and p<0.90 for Runx2. Achilles tendon: p<4.0E-04 for Coll. I; p<0.005 Tenom; p<0.01 for LPL; p<0.02 Sox9; and p<0.008 for Runx2). These increases were also significant when compared with the respective groups stretched to 4% (patellar tendon: p<0.003 for Coll. I; p<0.005 for Tenom; p<8.0E-04 for LPL; p<0.001 for Sox9; and p<0.009 for Runx2. Achilles tendon: p<1.0E-05 for Coll. I; p<0.35 for Tenom; p<0.002 for LPL; p<0.013 for Sox9; and p<0.015 for Runx2).

**Figure 9:**
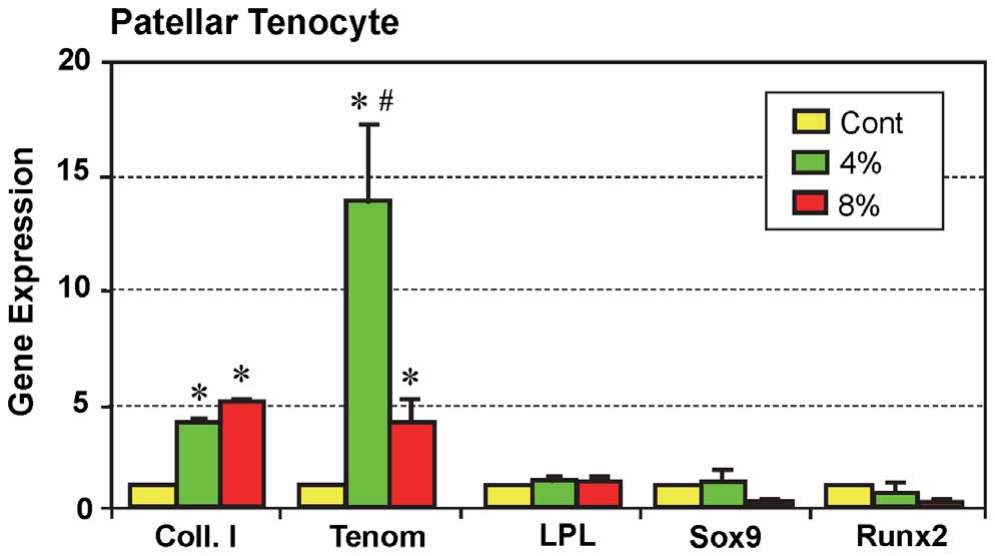
The effects of mechanical loading on gene expression in the mouse patellar tenocytes *in vitro*. Total RNA were collected from tenocytes stretched to 4% or 8% for qRT-PCR analysis. Both low (4%) and high (8%) mechanical stretching caused the expression of tenocyte-related genes (Coll. I, or Collagen type I; and Tenom or tenomodulin). However, regardless of the magnitude of the mechanical loading condition, the expression of non-tenocyte related genes LPL, Sox9, and Runx2 was not induced (*p<0.05, with respect to non-loaded cells; ^#^p<0.05 with respect to 8% stretching).

In contrast, only tenocyte-related genes were elevated in tenocytes derived from mouse patellar tendons and stretched to 4% (p<0.005 for Coll. I; and p<0.004 for Tenom ) or 8% (p<2.0E-04 for Coll. I; and p<0.004 for Tenom), while there was no increase in the expression of non-tenocyte-related genes (LPL, Sox9, and Runx2; Figure 9). However, Sox9 and Runx2 expression were lower in tenocytes stretched to 8% compared to the control (p<0.01 for Sox9; and p<0.033 for Runx2) and 4% stretching (p<0.13 for Sox9; and p<0.014 for Runx2). Interestingly, the expression of tenomodulin was significantly higher (p<0.006) after 4% stretching when compared to 8% stretching. Note that because growth of tenocytes from Achilles tendons was very slow, sufficient numbers of healthy cells could not be obtained for gene expression analysis and therefore these stretching experiments were not performed on Achilles tenocytes.

## Discussion

Tendons are constantly subjected to mechanical loading *in vivo*, which is known to alter tendon structure and function. Therefore, defining the effects of mechanical loading on tendons is essential to gain a better understanding of tendon patho-physiology. Using an *in vivo* model, we have shown that while ITR increased the vascularity in and around mouse patellar and Achilles tendons, MTR did not have an apparent effect. Previous studies also found increased vascularization in rat Achilles tendons following 12 weeks of uphill treadmill running [30] or in both patellar and Achilles tendons of rats on a 12 or 16 weeks downhill treadmill running regimen [31]. Therefore, it appears that tendons in rats undergo active repair and/or remodeling in response to such intensive, chronic mechanical loading conditions. In contrast, a recent study reported no change in the vascularity of Achilles tendons of rats that were on a long-term running regimen (12 weeks) when compared to control rats that did not run [32]. Possible reasons for this discrepancy include differences in the running protocols and the method of determining vascularization [30], [31].

In addition, we examined the “activity” of tendons in response to mechanical loading conditions by measuring the gene expression of MGF, an Eb form of IGF-1, which is a growth hormone that promotes tissue growth [33]. Our *in vivo* experiments showed that high levels of the MGF gene was expressed in both patellar and Achilles tendons after MTR, and more so after ITR. Similarly, uphill running for 12 weeks was recently reported to increase the expression of both MGF and the Ea isoform of IGF-1 in rat Achilles tendons [32]. Taken together, these results suggest the critical role of various IGF isoforms in mechanical load induced tissue repair. We also observed a 2-fold higher MGF level in the patellar tendons than Achilles tendons, suggesting the higher mechanical load and larger extent of “micro-injury” (e.g. “micro-tears” after repetitive mechanical loading [34]) on patellar tendons than on Achilles tendons during mouse treadmill running, which is a type of repetitive mechanical loading. An increase in the expression of MGF has been consistently observed during mechanical loading or repair of muscle tissue [35]. In this post-mitotic tissue, MGF significantly increased the number of progenitor cells in both healthy and diseased muscle, thus potentially facilitating their repair and maintenance [36]. Therefore, it is presumable that MGF upregulation in response to MTR and ITR could be significant for the tendon repair and/or remodeling under mechanical loading conditions.

There is little doubt that tendon’s biological response to mechanical loading is mainly caused by the tendon cells that include tenocytes and TSCs [10], [14]. In this study we showed that after MTR and ITR, higher numbers of quickly growing TSCs were present in mouse tendons when compared to tendons of control mice that did not run. The number of TSCs and their growth rate depended on loading intensity, with ITR generating more TSCs and inducing quicker cell proliferation than MTR. In a previous study a similar treadmill running also increased cell proliferation in rat Achilles tendons [37]. These findings indicate that more tendon cells, particularly TSCs, are generated for the repair and/or remodeling of tendons in response to the demands of mechanical loads placed on the tendons. Specifically, under mechanical loading conditions, TSC population in the tendon grows, providing progenitors for tenocytes and enhancing the remodeling of tendons. This may explain why appropriate exercise, like MTR, induces anabolic effects on the tendons, including enlarged cross-sectional area [2], increased tendon stiffness [3], and enhanced tendon tensile strength [4], [5].

In addition, we investigated the gene expression patterns in patellar and Achilles tendons of mice on treadmill. Both MTR and ITR induced the expression of tenocyte-related genes (collagen type I and tenomodulin). Since collagen type I is a major component of the tendon, its high levels of expression suggest that under both loading conditions, anabolic response of the tendon occurred. Our results are consistent with previous studies that reported a similar increase in collagen type I in tendons of rats under short-term training (4 days) [17]; however, others showed no change in collagen type I expression in rat tendons exposed to mechanical loading for 7 weeks [38] or 12 weeks [32], [39]. These discrepancies could be due to differences in experimental protocols particularly the method of mechanical loading. In a more recent study, ITR was shown to induce tendon remodeling in mice, by enhancing collagenous and non-collagenous protein synthesis and increasing the diameter and area of collagen fibrils [40]. Production of collagen I, a primary component of tendon matrices [41], also increased following exercise in humans [42].

Tenomodulin, a tenocyte marker [43], is thought to be involved in multiple aspects of tendon development, including tenocyte proliferation, collagen fibril maturation and organization, and possibly tendon vascularity [44]. Therefore, high levels of tenomodulin expression suggest the occurrence of a repair and/or remodeling event in the mouse tendons after MTR and ITR; this was particularly indicated by the enhanced vascularity in and near the mouse patellar and Achilles tendons under ITR conditions (Figure 1C, F). Collectively, these findings may explain the mechanobiological effects of MTR and ITR on tendon strength such as promoting tenogenesis, production of more collagen, and remodeling of tendon matrices.

We observed similar results in our *in vitro* study on patellar and Achilles TSCs which supported the findings with the *in vivo* model. In addition to the increase in cell proliferation and stem-cell related gene expression after mechanical loading, the *in vitro* study reported here shows that mouse TSCs, not tenocytes, are responsible for the upregulation of non-tenocyte-related genes LPL, Sox9, and Runx2 (specific markers for adipocytes, chondrocytes, and osteocytes, respectively) under ITR conditions. Similar response to mechanical loading was also observed in our previous study, which used rabbit TSCs instead of mouse TSCs in this study [14]. It should be noted that like all *in vitro* model systems, this model is also necessarily less physiologically accurate than an animal model because of the absence of other *in vivo* variables, such as extracellular matrix surrounding the tendon cells. For this very reason we used both *in vitro* and *in vivo* models to investigate the effects of mechanical loading on tendons in this study.

The finding that ITR elicited high levels of non-tenocyte gene expression suggests a potential TSC-based novel mechanism for the pathogenesis of degenerative tendinopathy; that is, mechanical over-loading induced upregulation of non-tenocyte-related genes results in tendinopathy. This causes TSCs to differentiate into non-tenocytes, leading to degenerative changes in affected tendons, which at later stages, manifest as lipid deposition, increased amounts of proteoglycans, and calcified tissue in the affected tendon [45]–[47]. This TSC-based mechanism of degenerative tendinopathy is also supported by the findings of previous studies. For example, increase in cartilage-related gene expression was observed in a rat tendon-overuse treadmill running model [48] and overuse of the rat supraspinatus tendon via treadmill running [49]. Additionally, in a rat model study, “round tenocytes” were observed in the supraspinatus tendon after intensive treadmill running (16.7 m/min) for 12 weeks [31]. Based on our findings in this study, we suspect that these “round tenocytes” could be chondrocytes differentiated from TSCs, because i) TSCs, not tenocytes, are able to undergo non-tenocyte differentiation under high mechanical loading conditions (Figs. 8, 9); ii) a round shape is a typical morphology of chondrocytes, and iii) these round cells produce abundant proteoglycans detected around the cells in the tendon [31].

In summary, this study was designed to define the effects of mechanical loading on tendons at the tissue and cellular levels using *in vivo* and *in vitro* models. The findings show that moderate mechanical loading, such as appropriate exercise, enhances tendon anabolism by upregulating MGF expression and stimulating TSC proliferation, both of which may be responsible for the exercise-induced changes in tendon structure that have been recognized for a long time. On the other hand, while excessive mechanical loading on tendons still enhanced MGF expression, TSC proliferation, and increased tenocyte-related gene expression, this kind of loading condition may also cause degenerative changes in tendons (*i.e.* degenerative tendinopathy) by inducing aberrant differentiation of TSCs into non-tenocytes (e.g. adipocytes, chondrocytes, and osteocytes).
